# The impact of a multimodal intervention on emergency department crowding and patient flow

**DOI:** 10.1186/s12245-019-0238-7

**Published:** 2019-08-27

**Authors:** M. C. (Christien) van der Linden, H. M. E. (Jet) van Ufford, Project Group Medical Specialists, Roeline A. Y. de Beaufort, Robert W. Grauss, Herman M. A. Hofstee, Jochem M. Hoogendoorn, Sven A. G. Meylaerts, Roselyne M. Rijsman, Theo P. W. de Rooij, Christiaan Smith, Frans J. de Voeght, Olga J. G. Warffemius, Geesje van Woerden, N. (Naomi) van der Linden

**Affiliations:** 1 Haaglanden Medical Center (HMC), P.O. Box 432, 2501 CK The Hague, the Netherlands; 2 HMC, P.O. Box 432, 2501 CK The Hague, the Netherlands; 3 Project Group Medical Specialists, HMC, P.O. Box 432, 2501 CK The Hague, the Netherlands; 4 Erasmus School of Health Policy and Management, Erasmus University Rotterdam, Rotterdam, the Netherlands

**Keywords:** Crowding, Emergency department, Staffing, Nurse practitioner, Patient flow, Quality of healthcare

## Abstract

**Objective:**

The objective of this study is to assess the impact of a multimodal intervention on emergency department (ED) crowding and patient flow in a Dutch level 1 trauma center.

**Methods:**

In this cross-sectional study, we compare ED crowding and patient flow between a 9-month pre-intervention period and a 9-month intervention period, during peak hours and overall (24/7). The multimodal intervention included (1) adding an emergency nurse practitioner (ENP) and (2) five medical specialists during peak hours to the 24/7 available emergency physicians (EPs), (3) a Lean programme to improve radiology turnaround times, and (4) extending the admission offices’ openings hours.

Crowding is measured with the modified National ED OverCrowding Score (mNEDOCS). Furthermore, radiology turnaround times, patients’ length of stay (LOS), proportion of patients leaving without being seen (LWBS) by a medical provider, and unscheduled representations are assessed.

**Results:**

The number of ED visits were grossly similar in the two periods during peak hours (15,558 ED visits in the pre-intervention period and 15,550 in the intervention period) and overall (31,891 ED visits in the pre-intervention period vs. 32,121 in the intervention period). During peak hours, ED crowding fell from 18.6% (pre-intervention period) to 3.5% (intervention period), radiology turnaround times decreased from an average of 91 min (interquartile range 45–256 min) to 50 min (IQR 30–106 min., *p* < 0.001) and LOS reduced with 13 min per patient from 167 to 154 min (*p* < 0.001). For surgery, neurology and cardiology patients, LOS reduced significantly (with 17 min, 25 min, and 8 min. respectively), while not changing for internal medicine patients. Overall, crowding, radiology turnaround times and LOS also decreased. Less patients LWBS in the intervention period (270 patients vs. 348 patients, *p* < 0.001) and less patients represented unscheduled within 1 week after the initial ED visit: 864 (2.7%) in the pre-intervention period vs. 645 (2.0%) patients in the intervention period, *p* < 0.001.

**Conclusions:**

In this hospital, a multimodal intervention successfully reduces crowding, radiology turnaround times, patients’ LOS, number of patients LWBS and the number of unscheduled return visits, suggesting improved ED processes. Further research is required on total costs of care and long-term effects.

## Background

Crowding in the emergency department (ED) is associated with lesser patient flow and adverse patient outcomes [1–3]. ED crowding is usually a consequence of insufficient inpatient hospital capacity or inadequate coordination of capacity within a hospital, and subsequently, the boarding of patients in the ED for extended periods of time [4, 5]. Experts widely agree that ED crowding is a system-wide problem, not one that solely results from problems in the ED or one that can be addressed using only ED-based interventions [6, 7]. Improving patient flow is essential to reduce ED crowding, but requires involvement of professionals outside the ED [8, 9] and support from hospital management. Therefore, a Taskforce Acute Care Team (TACT) was introduced in the study setting: a group of hospital managers and medical specialists dedicated to improve the quality of patient care and patient flow, addressing acute care as a strategic priority. The TACT introduced four process changes with the intention to improve patient flow at the ED: (1) adding an emergency nurse practitioner (ENP) and (2) adding five medical specialists during ED peak times (noon to 8 pm) to the 24/7 available emergency physicians (EPs), (3) a Lean programme to improve radiology report turnaround times and (4) extending the admission offices’ openings hours to help ED staff with finding an inpatient bed. This multimodal intervention was formally implemented in November 2017. The present study focuses on the impact of the multimodal intervention on ED crowding and patient flow.

In previous research, similar clinical redesign projects have been studied, including streaming patients directly from triage to dedicated minor injury units or super tracks [10] and ENP-led units [11–13], senior early assessment models of care [14–17], Lean methods [18–21], and improving access to inpatient admission [22]. Improving ED processes has been shown to reduce the length of stay (LOS), although studies show varying results, and the effect on crowding is uncertain [23].

The purpose of this study is to test the impact of a multimodal intervention on ED crowding and patient flow in a Dutch level 1 trauma center. In this study, we assess crowding levels, radiology report turnaround times and patients’ ED LOS during peak hours and overall. To ensure that enhanced patient flow is not achieved at the expense of high quality of care, we assess the percentage of patients leaving without being seen (LWBS) by a medical provider, reflecting potential harmful outcomes [24]. Furthermore, we describe the number of unscheduled representations to the ED within 1 week of the initial visit, a measure of patient safety [25].

## Methods

### Design

In this observational, cross-sectional study, we compare crowding, radiology turnaround times, patients’ ED LOS, and the proportion of patients LWBS and unscheduled representations between a 9 months pre-intervention period (December 2016 to September 2017) and the 9 months intervention period (December 2017 to September 2018).

### Setting

The study was performed at Haaglanden Medical Center (HMC) Westeinde, an inner-city, 380-bed acute neurovascular and level 1 trauma center in the Netherlands. The ED has an annual census of 54,000 adult and paediatric patient visits and a 24% admission rate. All patients are registered in the hospital database before they proceed to triage. After triage, patients who are eligible for treatment by a general practitioner (GP) are redirected to the GP cooperative (GPC), which is located next to the ED. The remaining patients are assessed at the ED. In this study, patients who are redirected to the GP are excluded from the analysis.

The nursing staff consists of 75% certified emergency nurses (CENs), 20% registered nurses in training for CEN, and 5% ENPs. Dutch ENPs are independent practitioners who are able to assess, diagnose, treat, prescribe, and refer to other health specialties. EPs are available 24/7. Per shift, one EP, one or two EPs in training, one surgical resident, one neurology resident, one internal medicine resident, one gynaecology resident, and one cardiology resident work at the ED. Attending specialists (non-EP) are available in the hospital (office hours) and on-call (out-of-hours) when needed. Imaging procedures are read by radiology residents who release a preliminary report through the Picture Archiving and Communication System (PACS) with a subsequent final attending report within 24 h.

### Intervention

In previous years, multiple process changes targeting ED crowding were successfully implemented in our ED including triage, a GPC, acute admission units [26], and 24/7 coverage of EPs. Nowadays, the ED has a relatively short LOS compared with nationally and internationally reported targets [27]. To prepare for expected future developments impacting acute care in our country, such as the closure of EDs and the increase of elderly patients presenting to the ED, the TACT aims to further improve ED patient flow. Their multimodal intervention included four process changes, intended to decrease crowding and improve ED patient flow:

1. One ENP was added to the nursing team per day shift and evening shift to improve flow for patients with minor injuries and minor illnesses who were not eligible for redirection to the GPC, e.g. patients with fractures of extremities.
2. Seven days a week, during peak hours, the ED medical staff was expanded with five attending medical specialists (cardiologist, internist, neurologist, radiologist, and surgeon), working side by side with the EPs, residents, and ENPs. The five attending medical specialists are involved in patients’ assessment on arrival and they perform direct on-site supervision. This change was trialled for 10 weeks in 2016 and showed an overall decrease in LOS with large variability by specialty [28, 29]. The residents kept working as usual. One EP per shift undertook a more coordinating role during the intervention period. This EP could initiate a team-based approach when indicated, with patients assessed and managed simultaneously by several attending specialists at an early stage.
3. A 5-day Lean project within the radiology department was organised, including key stakeholders such as the radiographers, radiology residents, radiologists, and management. In Lean methodology, the use for any goal other than the creation of value is wasteful and should be eliminated. In radiology, this translates into strategies to reduce wait times [18]. Our Lean team focused on improved radiography and report turnaround time, one of the largest bottlenecks in the ED process according to the attending specialists [28]. The Lean team introduced, amongst other strategies, a diagnostic fast-track for CT, a strategy to reduce back and forth phone calls, and the installation of runners for the transport of patients to and from ED and radiology room.
4. The admission offices’ openings hours were extended from 8 am to 5 pm into from 8 am to 8 pm, to better match the ED peak hours and thus, improve the outflow of the ED. Furthermore, the nurses from the inpatient units were asked to collect the patients from the ED within 30 min after the decision to admit.

### Outcome measures

The outcome of interest was ED crowding, measured with the modified National ED OverCrowding Score (mNEDOCS), a multidimensional scale to measure patient volume and throughput in hospitals [30]. The mNEDOCS alike the NEDOCS, is subdivided into 6 categories: 0–20, not busy; 21–60, busy; 61–100, extremely busy; 101–140, crowded; 141–180, severely crowded; > 180, disaster) [31]. Variables needed to calculate the mNEDOCS include total ED beds, total hospital beds, total patients in the ED, total admits in the ED, longest admit time in hours, waiting room time in hours of the most recent patient placed in a bed in the ED, and the number of patients who are being resuscitated or assigned the highest acuity level (Table 1). The mNEDOCS has been shown to correlate well with perceived crowding in this ED [30].

**Table 1:** Variables included in the NEDOCS and mNEDOCS

Total patients in ED—the number of patients in the ED, at the time the score is calculated. This includes patients in all areas including waiting patients.	
ED beds—the total number of ED beds that can be used to serve patients at the time the score is calculated.	
# admits—the number of holdovers/admits, in the ED, at the time the score is calculated.	
Total hospital beds—the total number of hospital beds.	
For NEDOCS: # vent patients in ED—the number of patients on ventilators/respirators in the ED at the time the score was calculated. For mNEDOCS: # critical care level 1 patients in ED—the number of patients who were being resuscitated or assigned the highest acuity level, at the time the score was calculated.	
Longest admit boarding time (in ED)—the longest admit holdover/boarding (in hours) at the time the score was calculated.	
Hrs longest wait in waiting room (last bed time)—the wait time (in hours) from arrival to bed for the last patient called for bed.	

Secondary outcomes were radiology report turnaround times, defined as the time from order entry to the time the radiology releases a report in the PACS system; patients’ ED LOS; the proportion of patients LWBS and the proportion of unscheduled representations within 1 week of the initial ED visit.

Outcome data were extracted from the clinical information system, as were demographic details (age, sex), and visit information (date and time of ED arrival, triage level, number and type of diagnostics requests, date and time of ED discharge, and discharge disposition).

### Analysis

Descriptive statistics were used to characterise the sample of the pre-intervention and intervention period. A computer programme queries the hospital information system for the data required to calculate the mNEDOCS at 15-min intervals and the scores during peak hours and overall were compared between the pre-intervention and intervention period. Radiology report turnaround times and LOS were calculated for patients arriving during peak times and for patients arriving 24/7 and compared between the pre-intervention and intervention period. We also calculated and compared total number, number of hospital admissions, and LOS of patients arriving during peak hours who were evaluated for (pre-intervention period) and by (intervention period) cardiology, surgery/traumatology, neurology, internal medicine, and other (patients who were assessed by one of the other medical specialties) and for patients who were having at least one radiology procedure.

Age, LOS, and mNEDOCS are reported as median (IQR). Differences between the periods were compared using the Mann-Whitney *U* test, given the non-normal distribution of the metrics. To compare categorical variables between the periods, we used *χ*^*2*^ tests. Significance threshold was set at a *p* value of 0.05. Statistical analysis was performed using SPSS (Statistical Package for the Social Sciences, IBM Corp., IBM SPSS Statistics for Windows, Version 22.0, Armonk, NY, USA).

### Ethics

The datasets did not contain individual identifiers to ensure the anonymity of the patients. The regional medical ethics committee and the institutional review board approved the study (Southwest Holland, nr. 17–122).

## Results

**Fig. 1:**
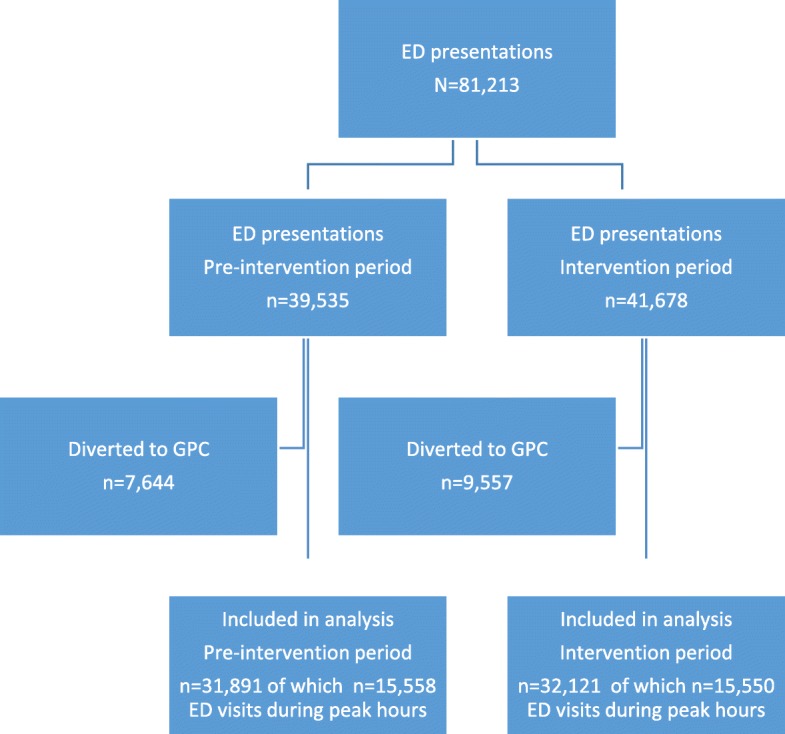
Flow diagram of patient presentations during study periods. Abbreviations: ED, emergency department; GPC, general practitioner cooperative

During the 18-month study period, 81,213 patient presentations were registered, 39,535 (48.7%) during the pre-intervention period, of which 7644 (19.3%) were diverted to the GPC, and 41,678 (51.3%) during the intervention period of which 9557 (22.9%) were diverted to the GPC. The remaining 64,012 patient presentations were included: 31,891 in the pre-intervention period and 32,121 in the intervention period (Fig. 1).

In the intervention period, there were more elderly patients and lesser children, lesser self-referred patients, lesser high-acuity but more urgent patients, lesser patients with limb problems and ear/nose/throat/eye problems, and more patients with shortness of breath and feeling unwell, compared to the pre-intervention period. There was no difference between the pre-intervention and intervention period in the number of patients arriving during peak hours (Table 2), and no differences in the number of patients needing hospital admission, patients who died at the ED and patients with one or more requests for imaging procedures during their ED visit (Table 3).

**Table 2:** Baseline characteristics of patient presentations and dispositions in the study periods

	Pre-intervention period *n* = 31,891	Intervention period *n* = 32,121	Odds ratio (95% CI)	*p* value^a^
Median age^b^ in years (IQR*)	45 (26–65)	47 (27–66)	1.00 (1.00–1.00)	< 0.001
Age in years^b^ *n* (%)				
17 years and younger	4273 (13.4)	4019 (12.5)	0.92 (0.88–0.97)	0.001
18–64 years	19,493 (61.1)	19,449 (60.5)	0.98 (0.95–1.01)	0.130
65–74 years	3694 (11.6)	3981 (12.4)	1.08 (1.03–1.13)	0.002
75 years and older	4426 (13.9)	4672 (14.5)	1.06 (1.01–1.10)	0.016
Self-referred	11,278 (35.4)	10,841 (33.8)	0.93 (0.90–0.96)	< 0.001
Acuity level^c^ *n* (%)				
Life-threatening	427 (1.3)	413 (1.3)	0.96 (0.84–1.10)	0.554
High urgent	7546 (23.7)	7351 (22.9)	0.96 (0.92–0.99)	0.020
Urgent	14,339 (45.0)	15,172 (47.2)	1.10 (1.06–1.13)	< 0.001
Standard	8663 (27.2)	7841 (24.4)	0.87 (0.84–0.90)	< 0.001
Non-urgent	230 (0.7)	214 (3.5)	0.92 (0.77–1.11)	0.402
Medical specialism *n* (%)				
Cardiology	4396 (13.8)	4454 (13.9)	(0.96–1.05)	0.764
Surgery/traumatology	9601 (30.1)	10,219 (31.8)	1.08 (1.05–1.12)	< 0.001
Neurology	3750 (11.8)	3785 (11.8)	1.00 (0.96–1.05)	0.923
Internal medicine	4493 (14.1)	4647 (14.5)	1.03 (0.99–1.08)	0.171
Other specialism^d^	9651 (30.3)	9016 (28.1)	0.90 (0.87–0.93)	< 0.001
Presenting problem^c^ *n* (%)				
Abdominal pain	4931 (15,8)	5071 (16.4)	1.04 (1.00–1.09)	0.053
Back pain	371 (1.2)	361 (1.2)	0.98 (0.85–1.13)	0.787
Chest pain and collapse	4353 (13.9)	4221 (13.6)	0.97 (0.93–1.02)	0.245
Ear/nose/throat/eye	682 (2.2)	601 (1.9)	0.89 (0.79–0.99)	0.032
Headache and head injury	2379 (7.6)	2248 (7.3)	0.95 (0.89–1.01)	0.082
Limb problems	5302 (17.0)	4829 (15.6)	0.90 (0.87–0.94)	< 0.001
Psychiatric disorders	1148 (3.7)	1153 (3.7)	1.01 (0.93–1.10)	0.773
Severe trauma and falls	2263 (7.2)	2262 (7.3)	1.01 (0.95–1.07)	0.806
Shortness of breath	2357 (7.6)	2537 (8.2)	1.09 (1.03–1.16)	0.003
Unwell patient	3629 (11.6)	3991 (12.9)	1.12 (1.07–1.18)	< 0.001
Wounds and local infections	2063 (6.6)	2008 (6.5)	0.98 (0.92–1.04)	0.519
Other presenting problem^e^	1736 (5.6)	1700 (5.5)	0.99 (0.92–1.06)	0.684
Arrival during peak hours (noon–8 pm)	15,115 (47.4)	15,195 (47.3)	1.00 (0.97–1.03)	0.819

*Abbreviation: *CI* confidence interval, *ED* emergency department, *IQR* interquartile range

^a^*p* values were calculated using *χ*^*2*^ tests, except for median age, which was calculated using the Mann-Whitney *U* test

^b^Based on 64,007 presentations, due to 5 missings

^c^Based on 62,196 presentations, due to 1816 (2.8%) missings

^d^Other specialism: all visits for other specialism besides cardiology, internal medicine, neurology, and surgery

^e^Other presenting problem: presenting problem occurring less than 600 times during the study periods: allergy, asthma, diabetes, facial problems, irritable child, neck pain, sexually acquired infections

**Table 3:** Patient dispositions in the study periods

	Pre-intervention period *n* = 31,891	Intervention period *n* = 32,121	Odds ratio (95% CI)	*p* value^a^
One or more imaging procedures requested	15,558 (48.8)	15,550 (48.4)	0.99 (0.96–1.02)	0.344
Hospital admission	8467 (26.5)	8620 (26.8)	(0.98–1.05)	0.413
Cardiology^b^	1742 (39.6)	1636 36.7)	0.88 (0.81–0.96)	0.005
Surgery/traumatology^c^	1407 (14.7)	1649 (16.1)	1.12 (1.04–1.21)	0.004
Neurology^d^	1714 (45.7)	1550 (41.0)	0.82 (0.75–0.90)	< 0.001
Internal medicine^e^	1376 (30.6)	1501 (32.3)	1.08 (0.99–1.18)	0.09
Other specialism^f^	2228 (23.1)	2284 (25.3)	1.13 (1.06–1.21)	< 0.001
Dead at the ED	39 (0.1)	50 (0.2)	1.27 (0.84–1.94)	0.257
Admission LOS^g^ in minutes (IQR)	3940 (1539–8676)	3576 (1364–7411)		< 0.001

*Abbreviation: *CI* confidence interval, *ED* emergency department, *IQR* interquartile range

^a^*p* values were calculated using *χ*^*2*^ tests, except for median admission LOS, which was calculated using the Mann-Whitney *U* test

^b^Based on 8850 cardiology visits, 4396 in the pre-intervention period and 4454 in the intervention period

^c^Based on 19,620 surgery/traumatology visits, 9601 in the pre-intervention period and 10,219 in the intervention period

^d^Based on 7535 neurology visits, 3750 in the pre-intervention period and 3785 in the intervention period

^e^Based on 9140 internal medicine visits, 4493 in the pre-intervention period and 4647 in the intervention period

^f^Other specialism: all visits for other specialism besides cardiology, internal medicine, neurology, and surgery, based on 18,667 visits, 9651 in the pre-intervention period and 9016 in the intervention period

^g^Based on 16,475 admissions (8388 in the control period and 8087 in the intervention period)

During the intervention period, significantly less plain radiographs but more CT-scans, ultrasounds, and other radiology orders were executed (Table 4). These findings were similar when focusing on the peak hours of the study periods (data not shown).

**Table 4:** Radiology orders during the study periods

Categories of imaging procedures *n* (%)^a^	Pre-intervention period *n* = 20,080	Intervention period *n* = 20,590	Odds ratio (95% CI)	*p* value^b^
Plain radiograph	11,327 (56.4)	10,962 (53.0)	0.87 (0.84–0.91)	< 0.001
CT^*^	6094 (30.3)	6670 (32.2)	1.09 (1.05–1.14)	< 0.001
Ultrasonography	2511 (12.5)	2799 (13.5)	1.10 (1.03–1.16)	0.002
Other imaging procedure^c^	150 (0.7)	256 (1.2)	1.67 (1.36–2.04)	< 0.001

*Abbreviation: *CT* computer tomography

^a^Based on 40,670 orders, due to 99 missings

^b^*p* value was calculated using *χ*^*2*^ test

^c^Other imaging procedures includes magnetic resonance imaging and radiograph with contrast

### Crowding

The mNEDOCS was calculated each 15 min of every hour. The computerised system broke down during 41 days from 19 January 2018 until 28 February 2018, resulting in the loss of 3936 scores. In the control period, 379 scores were omitted due to system problems. All missing measurements in one of the two periods were deleted in the other one, matched on date and time, resulting in a data file with the remaining 43,978 scores, 21,989 in the pre-intervention period and 21,989 in the intervention period.

During peak times, the measurements of mNEDOCS above 100 (indicating crowding) fell from 18.6% in the pre-intervention period to 3.5% in the intervention period. It was significantly more often “not busy” or “busy,” indicated by a mNDEDOCS of 60 and lower (30.8% of the measurements during the pre-intervention period and 60.9% of the measurements during the intervention period (Table 5)).

**Table 5:** Crowding measurements during peak hours (noon to 8 pm) of the study periods

mNEDOCS measurements	Pre-intervention period *n* = 7335	Intervention period *n* = 7335	Odds ratio (95% CI)	*p* value^a^
Median mNEDOCS (IQR)*	74 (56–94)	54 (38–71)	0.97 (0.97–0.97)	< 0.001
Crowding (mNEDOCS > 100) *n* (%)	1367 (18.6)	260 (3.5)	0.16 (0.14–0.18)	< 0.001
mNEDOCS categories *n* (%)				
0–20, not busy	111 (1.5)	500 (6.8)	4.80 (3.87–5.86)	< 0.001
21–60, busy	2150 (29.3)	3969 (54.1)	2.84 (2.66–3.04)	< 0.001
61–100, extremely busy	3707 (50.5)	2606 (35.5)	0.54 (0.51–0.58)	< 0.001
101–140, crowded	1226 (16.7)	257 (3.5)	0.18 (0.16–0.21)	< 0.001
141–180, severely crowded	138 (1.9)	3 (0.0)	0.02 (0.01–0.07)	< 0.001
> 180, disaster	3 (0.0)	0 (0.0)	0.50 (0.49–0.51)	0.248

*Abbreviation: *IQR* interquartile range

^a^*p* values were calculated using *χ*^*2*^ tests, except for median mNEDOCS, which was calculated using the Mann-Whitney *U* test, and disaster, which was calculated using the Fisher exact test

Overall, 7.7% of the measurements in the pre-intervention period were above 100, while this was only 1.3% during the intervention period (Table 6). The ED was significantly less often ‘extremely busy’ (25.5% during the pre-intervention period vs. 15.5% during the intervention period (*p* < 0.001)).

**Table 6:** Overall (24/7) crowding measurements during the study periods

mNEDOCS measurements	Pre-intervention period *n* = 21,989	Intervention period *n* = 21,989	Odds ratio (95% CI)	*p* value^a^
Median mNEDOCS (IQR)*	41 (10–70)	28 (8–51)	0.99 (0.99–0.99)	< 0.001
Crowding (mNEDOCS > 100) n (%)	1688 (7.7)	292 (1.3)	0.16 (0.14–0.18)	< 0.001
mNEDOCS categories *n* (%)				
0–20, not busy	7354 (33.4)	8930 (40.6)	1.36 (1.31–1.42)	< 0.001
21–60, busy	7413 (33.7)	9369 (42.6)	1.46 (1.40–1.52)	< 0.001
61–100, extremely busy	5534 (25.2)	3398 (15.5)	0.54 (0.52–0.57)	< 0.001
101–140, crowded	1525 (6.9)	288 (1.3)	0.18 (0.16–0.20)	< 0.001
141–180, severely crowded	159 (0.7)	4 (0.0)	0.03 (0.01–0.07)	< 0.001
> 180, disaster	4 (0.0)	0	0.50 (0.50–0.51)	0.125

*Abbreviation: *IQR* interquartile range

^a^*p* values were calculated using *χ*^*2*^ tests, except for median mNEDOCS, which was calculated using the Mann-Whitney *U* test, and disaster, which was calculated using the Fisher exact test

### Radiology turnaround times

The median turnaround times for radiology reporting decreased significantly for plain imaging procedures, as well as for CT and ultrasonography during peak hours (Table 7) and overall (Table 8).

**Table 7:** Radiology turnaround times during the peak hours of the study periods

	Pre-intervention period, 10,564 orders in 8070 presentations	Intervention period, 10,990 orders in 7957 presentations	*p* value^b^
Median turnaround time radiology in minutes (IQR)^a^	71 (40–129)	38 (25–61)	< 0.001
Median turnaround time per category in minutes (IQR)^a^			
Plain radiograph	55 (32–96)	30 (21–45)	< 0.001
CT	94 (54–175)	56 (37–85)	< 0.001
Ultrasonography	109 (70–170)	37 (25–56)	< 0.001

*Abbreviation: *CT* computer tomography, *IQR* interquartile range

^a^Based on orders, due to missings

^b^*p* value was calculated using Mann-Whitney *U* tests

**Table 8:** Overall (24/7) radiology report turnaround times during the study periods

	Pre-intervention period, 20,082 orders in 15,558 presentations	Intervention period, 20,687 orders in 15,550 presentations	*p* value^b^
Median turnaround time radiology in minutes (IQR)^a^	91 (45–256)	50 (30–106)	< 0.001
Median turnaround time per category in minutes (IQR)^a^			
Plain radiograph	69 (36–195)	41 (25–83)	< 0.001
CT	122 (61–413)	67 (42–142)	< 0.001
Ultrasonography	113 (69–199)	48 (30–84)	< 0.001

*Abbreviation: *CT* computer tomography, *IQR* interquartile range

^a^Based on 40,670 orders, due to 99 missings

^b^*p* value was calculated using Mann-Whitney *U* tests

### Patients’ length of stay at the ED

During the peak hours, the median LOS reduced with 13 min from 167 to 154 min (*p* < 0.001). For surgery patients, LOS reduced with 17 min from 141 min to 124 min (*p* < 0.001) and for neurology patients, with 25 min from 203 min to 178 min (*p* < 0.001). The LOS for cardiology patients decreased by 8 min from 166 min to 158 min during peak hours. The LOS for internal medicine patients did not change (Table 9).

**Table 9:** Length of stay during the peak hours of the study periods

	Pre-intervention period *n* = 15,115	Intervention period *n* = 15,195	*p* value^b^
Median LOS in minutes (IQR)^a,*^	167 (113–236)	154 (100–220)	< 0.001
Median LOS per specialism in minutes (IQR)^a^			
Cardiology	166 (129–218)	158 (122–214)	< 0.001
Internal medicine	206 (151–282)	206 (149–274)	0.233
Neurology	203 (143–276)	178 (123–248)	< 0.001
Surgery	141 (93–208)	124 (79–183)	< 0.001
Other specialism^c^	160 (100–229)	147 (91–213)	< 0.001
Median LOS for self-referred patients (IQR)^d^	142 (94–203)	130 (84–191)	< 0.001
Median LOS for admitted patients (IQR)^e^	212 (155–287)	204 (147–271)	< 0.001
Cardiology	185 (142–246)	186 (143–251)	0.714
Internal medicine	240 (185–311)	240 (191–303)	0.789
Neurology	204 (141–287)	193 (131–275)	0.016
Surgery	230 (168–300)	196 (143–265)	< 0.001
Other specialism^c^	210 (154–287)	196 (141–253)	< 0.001
Median LOS for patients who had at least one imaging procedure^f^	194 (138–266)	180 (126–248)	< 0.001

*Abbreviation: *ED* emergency department, *LOS* length of stay, *IQR* interquartile range

^a^All LOS calculations are based on 30,208 presentations, due to 102 missings

^b^*p* value was calculated using Mann-Whitney *U* tests

^c^Other specialism; all visits for other specialism besides cardiology, internal medicine, neurology, and surgery (*n* = 7739)

^d^*n* = 8485

^e^*n* = 8261

^f^*n* = 16,027

Overall, the median LOS reduced with 8 min from 157 to 149 min (*p* < 0.001). While LOS for surgical patients and neurology patients decreased with 12 and 16 min, respectively, LOS for cardiology patients and internal medicine patients remained similar (Table 10).

**Table 10:** Overall (24/7) length of stay during the study periods

	Pre-intervention period *n* = 31,891	Intervention period *n* = 32,121	*p* value^b^
Median LOS in minutes (IQR)^a,*^	157 (102–228)	149 (95–218)	< 0.001
Median LOS per specialism in minutes (IQR)^a^			
Cardiology	164 (126–215)	160 (123–218)	0.056
Internal medicine	205 (145–285)	205 (145–279)	0.373
Neurology	191 (135–266)	175 (120–246)	< 0.001
Surgery	136 (89–203)	124 (79–187)	< 0.001
Other specialism^c^	137 (80–209)	132 (75–199)	< 0.001
Median LOS for self-referred patients (IQR)^d^	131 (84–192)	124 (78–184)	< 0.001
Median LOS for admitted patients (IQR)^e^	203 (146–277)	198 (140–269)	< 0.001
Cardiology	183 (140–237)	180 (137–244)	0.683
Internal medicine	238 (179–310)	241 (187–310)	0.342
Neurology	192 (131–270)	186 (124–267)	0.032
Surgery	215 (148–296)	196 (130–268)	< 0.001
Other specialism^c^	201 (143–276)	190 (133–254)	< 0.001
Median LOS for patients who had at least one imaging procedure^f^	191 (134–263)	181 (126–251)	< 0.001

*Abbreviation: *ED* emergency department, *LOS* length of stay, *IQR* interquartile range

^a^LOS calculations are based on 63,860 presentations, due to 152 missings

^b^*p* value was calculated using Mann-Whitney *U* tests

^c^Other specialism; all visits for other specialism besides cardiology, internal medicine, neurology, and surgery (*n* = 18,667)

^d^*n* = 22,119

^e^n = 17,087

^f^*n* = 31,108

### Patients leaving without being seen and unscheduled representations within 1 week

Significantly less patients LWBS: 348 patients in the pre-intervention period vs. 270 patients in the intervention period, *p* < 0.001. There were also significantly less unscheduled representations at the ED within 1 week after the initial ED visit: 864 (2.7%) in the pre-intervention period vs. 645 (2.0%) patients in the intervention period, *p* < 0.001.

## Discussion

This study demonstrates improvement in ED flow after the introduction of the multimodal intervention. Hours of crowding decreased significantly, as did the patients’ ED LOS. During the peak hours in the intervention period, we found a 13-min reduction in ED LOS per patient, totaling 3369 h between noon and 8 pm during the 274 day-intervention period. This additional time is now available for direct patient care.

The process changes were based on previous research: implementing care by ENPs can reduce waiting times in the ED [11–13]. Involvement of medical specialists is associated with increased decisiveness resulting in decreased LOS [17]. In a pilot study performed in the study setting, a decrease in LOS for admitted patients was found when medical specialists are involved at the ED. In that study, delays in radiology turnaround times were considered a main constraint for ED throughput [28]. This finding led to the organisation of a 5-day Lean project within the radiology department, focusing on improved turnaround time for radiography. Alike shown in other studies [18, 20], the Lean methodology was effective in reducing turnaround times for radiology results. Unfortunately, this reduction in waiting time for radiology results is only partially translated into reduced overall LOS. This is not surprising, since LOS is affected by factors beyond the ED process [20].

Our findings are in line with other studies showing that hospital-wide interventions reduce ED crowding [7, 9, 12]. The support of our hospital leadership and the TACT towards improving patient care was a mitigating factor in the success of the process changes. Bringing together diverse disciplines including medical specialists and ED staff fostered a new focus on ED flow as being not only an ED issue but a system-wide issue. Our multimodal intervention alleviated ED crowding and reduced ED patients’ LOS. Crowded circumstances (mNEDOCS > 100) decreased significantly. It seems plausible that the additional medical specialists available at the ED during peak times were (at least in part) responsible for this. When attending specialists make immediate assessments in the ED, there is less replication of work and decisions about next steps are made more quickly [32]. Additionally, the intervention enhanced focus on acute care throughout the hospital. This was an important driving factor in freeing hospital beds on behalf of acute admissions.

From the current research, it cannot be concluded whether additional attending specialists and ENPs were needed to obtain these results, or whether additional EPs or other human resources would have generated the same or even better effects. Adding attending specialists to the ED staff during peak hours is a costly intervention, while for some patient groups (e.g. internal medicine), there was no change in ED LOS. The total costs and cost-effectiveness of the current intervention and alternatives need to be compared. To study the cost-effectiveness of alternative staffing decisions, ED simulation modelling may be valuable.

A reason why ED LOS did not reduce for internal medicine patients might be that an internist had been available for the acute admission unit and the ED for many years already, focusing on ED outflow far before the current project started. Also, confounding factors may have played an important role. For example, the flu season lasted until the end of February during the intervention period, while it ended a month earlier during the pre-intervention period.

Our positive findings of decreased crowding and reduced patients’ LOS cannot be attributed to differences between the pre-intervention and intervention population. There was a small (0.7%) increase in the number of presentations and no difference in the number of hospital admissions. The differences between the populations suggest a sicker population in the intervention period: there were more elderly patients and patients with shortness of breath and feeling unwell, and lesser patients with limb problems and ear-nose-throat-eye problems. The first two groups are usually more labour-intensive for ED staff and patients have lengthier stays compared to patients with limb or ear/nose/throat or eye problems. Since the patient population in the intervention period was at least as high complex as the patient population in the pre-intervention period, the main reason for the significant decrease in crowding levels during the intervention period is probably the shortening of LOS. Shorter LOS is associated with improvements in patient experience [33] and a reduction of adverse events [34].

### Limitations

This study conveys the experience of a single institution and may have limited generalizability because of differences in patient population, level of crowding, and health care system. Some of the process changes and results may not be applicable to EDs working under different constraints. However, we feel that the process changes would be beneficial to many EDs.

One major factor which could have influenced crowding was a 3-year period of restructuring the ED which continued until mid-2018, halfway the intervention period. This was a six-phase construction project, each phase involving closing work areas and opening new spaces. Although the number of available treatment rooms remained similar throughout the study periods, ED staff may have experienced more frustration with patient care in a noisy environment during the pre-intervention period.

The intervention being studied was multimodal and ongoing during the intervention period, precluding separating the effects of each part of the intervention on the outcomes. We cannot attribute changes in ED crowding and LOS to the introduction of the intervention as it is impossible to control for other influences taking place simultaneously. Designing a randomised controlled trial to assess the effects of each part of the intervention was not ethical because process improvements should not be withheld from a part of the population. In a future study in another setting, it would be interesting to separate the effects of each change.

There may have been some variability in what patients and how many patients were evaluated and treated by the medical specialists and the ENPs, depending on the providers working and their level of training and experience with ED patients. Moreover, during the intervention period, ED staff and medical specialists knew that they were under observation, thereby potentially introducing a Hawthorne effect (change in behaviour induced by the study itself). However, it is expected that such an effect would not last 9 months.

We did not assess other staff models nor did we carry out an economic assessment of the interventions and their impact—a recommendation for future studies.

Regarding the number of patients with unscheduled return visits, it is possible that patients visited another ED which may have led to some cases not identified. However, it is unlikely that this would occur in only one of the periods.

A future study is needed to assess the effects of our multimodal intervention beyond the ED. For example, our findings show a decrease in presentations of unscheduled return visits within 1 week. It is possible that the number of scheduled revisits to the ED or to the policlinics also decreases when a medical specialist assesses the patient or when the radiologist already performs an ultrasound to exclude a certain diagnosis instead of rescheduling that patient the next day. It is also possible that medical specialists who experience difficulties in admitting a patient during their ED shift because of lack of beds are keener to discharge their inpatients before noon in the future, enhancing inpatient flow and decreasing access block.

## Conclusions

In this hospital, a multimodal intervention successfully reduced crowding, radiology turnaround times, patients’ LOS, number of patients LWBS, and the number of unscheduled return visits, suggesting improved ED processes. Further research is required on total costs of care and long-term effects.

## Data Availability

The datasets used and analysed during the current study are available from the corresponding author on reasonable request.

## Abbreviations

CEN: Certified emergency nurse
CI: Confidence interval
CT: Computer tomography
ED: Emergency department
ENP: Emergency nurse practitioner
EP: Emergency physician
GP: General practitioner
GPC: General Practitioner Cooperative
HMC: Haaglanden Medical Center
IQR: Interquartile range
LOS: Length of stay
LWBS: Left without being seen
mNEDOCS: Modified National Emergency Department OverCrowding Score
NEDOCS: National Emergency Department OverCrowding Score
PACS: Picture Archiving and Communication System
SPSS: Statistical Package of the Social Sciences
TACT: Taskforce Acute Care Team
TAT: Turnaround time
